# Diet-Induced Obesity Is Associated with an Impaired NK Cell Function and an Increased Colon Cancer Incidence

**DOI:** 10.1155/2017/4297025

**Published:** 2017-03-05

**Authors:** Ina Bähr, Vincent Goritz, Henriette Doberstein, Grit Gesine Ruth Hiller, Philip Rosenstock, Janine Jahn, Ole Pörtner, Tobias Berreis, Thomas Mueller, Julia Spielmann, Heike Kielstein

**Affiliations:** ^1^Institute of Anatomy and Cell Biology, Faculty of Medicine, Martin Luther University Halle-Wittenberg, Halle (Saale), Germany; ^2^Institute of Pathology, University Hospital of Martin Luther University Halle-Wittenberg, Halle (Saale), Germany; ^3^Clinic for Internal Medicine IV, Department of Hematology and Oncology, University Hospital of Martin Luther University Halle-Wittenberg, Halle (Saale), Germany

## Abstract

Obesity is associated with an increased colon cancer incidence, but underlying mechanisms remained unclear. Previous studies showed altered Natural killer (NK) cell functions in obese individuals. Therefore, we studied the impact of an impaired NK cell functionality on the increased colon cancer risk in obesity. In vitro investigations demonstrated a decreased IFN-*γ* secretion and cytotoxicity of human NK cells against colon tumor cells after NK cell preincubation with the adipokine leptin. In addition, leptin incubation decreased the expression of activating NK cell receptors. In animal studies, colon cancer growth was induced by injection of azoxymethane (AOM) in normal weight and diet-induced obese rats. Body weight and visceral fat mass were increased in obese animals compared to normal weight rats. AOM-treated obese rats showed an increased quantity, size, and weight of colon tumors compared to the normal weight tumor group. Immunohistochemical analyses demonstrated a decreased number of NK cells in spleen and liver in obesity. Additionally, the expression levels of activating NK cell receptors were lower in spleen and liver of obese rats. The results show for the first time that the decreased number and impaired NK cell function may be one cause for the higher colon cancer risk in obesity.

## 1. Introduction

Obesity is one of the most serious and escalating public health problems affecting all age and socioeconomic groups in developed as well as developing countries. In 2014, the World Health Organization reported that over 1.9 billion adults (39%) were overweight and more than 600 million adults (13%) were obese [1]. Obesity is associated with an increased risk and mortality rate for many serious diseases like type 2 diabetes, coronary heart disease, stroke, osteoarthritis, and several cancer types, like breast, kidney, liver, and colorectal cancer [1–3]. It has been established that up to 20% of all cancers can be contributed to obesity, including colon cancer, which is one of the prevalent forms of cancer worldwide [4, 5]. Recent studies had shown that with each five kg increase in body weight gain the colon cancer incidence was enhanced by 6% [6, 7]. In addition, high body mass index (BMI) in colon cancer patients was associated with an increased mortality rate [3, 8]. Although some obesity-related metabolic factors like adipocytokine levels, insulin resistance, intestinal microbiota, and chronic inflammation are believed to relate obesity and cancer, the underlying pathophysiological mechanisms linking obesity and cancer still remained unresolved [9, 10].

Natural killer (NK) cells are a major component of the innate immune system rapidly responding against virus-infected and tumor cells. On the one hand, NK cells mediate their antitumor response by direct cellular regulation of target cell activity via activating and inhibitory receptors as well as induction of target cell lysis via exocytosis of granzymes and perforin. On the other hand, NK cells activate the adaptive immune system by secreting different cytokines, like interferon-*γ* (IFN-*γ*) and tumor necrosis factor *α* (TNF-*α*), in order to regulate tumor progression and metastases [11].

Previous animal experiments demonstrated that NK cell functionality is disturbed in obesity [12–14]. The decreased NK cell activity was associated with an impaired leptin-dependent signal transduction in NK cells in obese rats [15]. Corresponding to animal experiments, studies on human individuals similarly showed a NK cell dysfunction in obesity [16–18]. Interestingly, the disturbed NK cell functionality could be ameliorated by transfer of NK cells to a normal weight metabolic environment in rats [19]. Furthermore, the altered NK cell activity could be reactivated after weight loss and fat mass reduction after bariatric surgery or a combined exercise and dietary program [20, 21].

Obesity is associated with increased plasma leptin concentrations of 30–40 ng/mL compared to 5–10 ng/mL plasma leptin concentrations in normal weight individuals [22, 23]. Recent studies had demonstrated that the NK cells express leptin receptors [15, 24]. In addition, expressions of leptin receptors on NK cells as well as postreceptor leptin signaling cascades were found to be impaired in obesity [15, 18, 24]. These data indicate a direct association on adipocytokine-mediated effects on NK cell functionality.

As the biological mechanisms for the elevated cancer incidence in overweight individuals still remained unclear, the aim of this study was to characterize the relationship between an altered NK cell functionality and the increased colon cancer risk in obesity.

## 2. Material and Methods

### 2.1. Cell Lines and NK Cell Isolation

Human colon adenocarcinoma DLD-1 cells were maintained in RPMI 1640 supplemented with 10% fetal bovine serum (FBS, both from Biochrom AG, Berlin, Germany), 100 U/mL penicillin, and 100 mg/mL streptomycin (both from Sigma-Aldrich, St. Louis, USA). The human NK cell line NK-92 was kindly provided by Prof. Dr. Roland Jacobs (Hannover Medical School, Hannover, Germany). Human primary NK cells were isolated from leukocyte filters obtained from the Institute for Transfusion Medicine, University Hospital, Halle (Saale), Germany. Peripheral blood mononuclear cells (PBMCs) from enriched leukocytes of buffy coats were separated by density gradient centrifugation on biocoll separating solution (Biochrom AG). Primary human NK cells were purified by negative magnetic separation using an NK cell isolation kit (Miltenyi Biotec, Auburn, CA, USA), according to the manufacturer's protocol. Isolated NK cells and NK-92 cells were cultivated in RPMI 1640 medium supplemented with 10% FBS, 100 U/mL penicillin, 100 mg/mL streptomycin, 1 mM sodium pyruvate, 2 mM L-glutamine (both from Biochrom AG), and 200 U/mL human recombinant interleukin- (IL-) 2 (Novartis AG, Basel, Switzerland).

### 2.2. Leptin Incubation, Cytotoxicity Assays, and IFN-*γ* Secretion of NK Cells

For molecular investigations, NK-92 cells either remained unstimulated or were preincubated with 10 ng/mL (physiological concentration in normal weight individuals) and 100 ng/mL (pathophysiological concentration in obese individuals) recombinant human leptin (R&D Systems, Minneapolis, MN, USA) for 4 h or 24 h. Cells were collected and stored at −80°C until analysis. The cytotoxicity of NK cells was analyzed using the DELFIA EuTDA Cytotoxicity kit (PerkinElmer, Waltham, MA, USA) according to the manufacturer's manual. NK-92 cells as well as primary NK cells served as effector cells and DLD-1 cells served as target cells. NK effector cells either remained unstimulated or were preincubated with 10 ng/mL and 100 ng/mL recombinant human leptin for 4 h or 72 h. To determine the cytotoxicity, NK cells were coincubated with DLD-1 cells for 1 h in RPMI 1640 medium supplemented with 10% FBS. Fluorescence data were recorded using a time resolved fluorometer (Synergy Mx, BioTek Instruments, Winooski, VT, USA). Remaining supernatants of the cytotoxicity assay were collected for IFN-*γ* analyses by luminex immunoassay (eBioscience, Frankfurt am Main, Germany). In both incubation experiments with leptin as well as cytotoxicity assays including analyses of IFN-*γ* secretion, the incubation medium of NK-92 and primary NK cells contained 200 U/mL IL-2.

### 2.3. Animal Experiments

Six-week-old male Wistar rats (*n* = 50) were obtained from Charles River GmbH (Sulzfeld, Germany) and were housed individually on a 12 : 12 light : dark cycle with free access to water and pelleted food. After an acclimatization period of one week, rats were randomized into two groups. One group (*n* = 25) received a normocaloric diet (control, 4% fat, C1090-10, Altromin, Lage, Germany) and the other group (*n* = 25) a high-fat high caloric diet (diet-induced obesity, DIO, 34% fat, C1090-60, Altromin) for 46 weeks. Eight weeks after start of feeding, eleven animals of each group were treated with azoxymethane (AOM; s.c. 15 mg/kg body weight; Sigma-Aldrich) to induce colon cancer growth in animals of the AOM groups or a subcutaneous control injection of 0.9% NaCl once a week for two weeks. Daily intake of energy, fat, protein, and carbohydrate was calculated using the daily food intake and data of diet composition given by the manufacturer (Altromin). 37 weeks after the last AOM injection, animals were anesthetized with isoflurane, final body weight was determined, and blood was sampled by heart ventricle puncture. Visceral (epididymal, mesenteric, and omental) fat mass was calculated and tissue samples of liver and spleen were preserved. The large intestine (from cecum to anus) was opened longitudinally and divided into two equal segments (proximal and distal). Colon was washed free of contents with ice-cold saline and pinned on a cork mat to examine for macroscopically visible tumors. Localization, size, weight, and distribution of all tumors were recorded. Tumor-bearing areas were excised and embedded in paraffin. All research and animal care procedures had been approved by the local Animal Care Committee.

### 2.4. Definition and Classification of Colon Tumors

Paraffin sections (5 *μ*m) of colon tumors were obtained and stained with hematoxylin and eosin. Tissue sections were analyzed in blind and classified according to histologic grade by an expert pathologist. Colon tumors induced by AOM were classified as adenomas or adenocarcinomas. Adenomas were defined histologically as lesions in which neoplastic cells were confined to the mucosal layer, while adenocarcinomas were defined as lesions in which neoplastic cells had penetrated the muscularis mucosa to involve the submucosa or deeper layers.

### 2.5. PBMC Isolation and Flow Cytometric Analysis

Rat blood PBMCs were separated by density gradient centrifugation as described above. Fluorescence-activated cell sorter (FACS) analyses were performed as described earlier [21]. The following mouse anti-rat antibodies were used: CD3 APC, CD4 PE-Cy 7, CD8a PE, CD45RA FITC, and CD161a PE (BD Biosciences, San Diego, USA). NK cells were represented by CD161a^bright^/CD3^−^ lymphocytes. Cells were measured with a LSR-Fortessa FACS analyzer and data were analyzed using BD FACSDiva Flow Cytometry Software (BD Biosciences).

### 2.6. Analysis of Serum Metabolites and Plasma Cytokines

The serum metabolites triacylglycerol, total cholesterol, low-density lipoprotein (LDL) cholesterol, and high-density lipoprotein (HDL) cholesterol were measured by a standard autoanalysis technique (Beckmann Coulter DxC 800 System, Beckman Coulter GmbH, Krefeld, Germany). Plasma concentrations of the cytokines leptin, TNF-*α*, interleukin- (IL-) 10, und IL-1*β* were quantified using a Multiplex Immunoassay (eBioscience) following the manufacturer's instructions. Data were analyzed using the Procartaplex-analyst 1.0 software (eBioscience).

### 2.7. Molecular Analysis

Total RNA of frozen spleen and liver tissue as well as NK-92 cells was extracted using Precellys 24 (Peqlab, Erlangen, Germany) and a Trizol-based extraction method (Sigma-Aldrich) according to the manufacturer's instructions. The RNA was quantified and its integrity was checked by agarose gel electrophoresis. After DNase treatment (Promega, Madison, WI, USA), cDNA synthesis was performed by reverse transcriptase reaction according to the supplier's instruction (Thermo Fisher Scientific Inc., Waltham, USA). The mRNA concentrations of genes were measured by real-time polymerase chain reactions (iQ5, BioRad, München, Germany) using SYBR® Green Fluorescein Mix (BioRad). Specific primers and sequences are listed in Table 1. For normalization of target gene values, the housekeeping genes Ppia (peptidylprolyl isomerase A, cyclophilin A for spleen and liver tissue) and (*β*-actin for NK-92 cells) were used as a reference throughout the experiments. The relative mRNA concentration was calculated using the ΔΔCt method [25] and individual amplification efficiency for each primer, determined by a standard curve with different primer dilutions.

### 2.8. Immunohistochemical Analyses

For characterization of NK cells in spleen and liver, 14 *μ*m thick cryostat sections were cut and placed on glass slides. Every third section was selected for immunohistochemical analyses performed with the alkaline phosphatase antialkaline phosphatase technique as previously described [15]. The primary mouse anti-rat antibody directed against the NK-RP1 receptor (CD 161, clone 10/78, AbD Serotec, Puchheim, Germany, 1 : 1000) was used. The sections were stained with Fast Blue (spleen) or Fast Red (liver; Fast Blue/Red TR Salt; Sigma-Aldrich), counterstained with hematoxylin (Mayer's hemalaun solution; Merck, Darmstadt, Germany) for 15 s, and mounted in glycergel (Dako Cytomation, Glostrup, Denmark). For the quantitative analysis in the liver and spleen of six animals per group, five sections and eight visual fields per section were examined for each animal (resulting in 38,4 m^2^ investigated area/group/organ). In the spleen sections, four visual fields in the red pulp and four fields in the transition zone between white and red pulp were selected. The quantitative immunohistological analyses were performed blinded to the treatment conditions using the Image J software (US National Institutes of Health, Bethesda, MD, USA).

### 2.9. Statistical Analysis

Statistical analyses were performed using unpaired *t*-test for cell incubation experiments or one-way ANOVA with the Tukey multiple comparison test for post hoc analysis for animal experiments. To analyze the effect of AOM treatment and the high-fat diet independently from each other on the different parameters, two-way ANOVA was applied. Differences were considered significant if *P* < 0.05. The software used was GraphPad Prism 7 (GraphPad Software, Inc., La Jolla, USA).

## 3. Results

### 3.1. Cytotoxicity Assay and IFN-*γ* Secretion of NK-92 and Primary Cells

**Figure 1:**
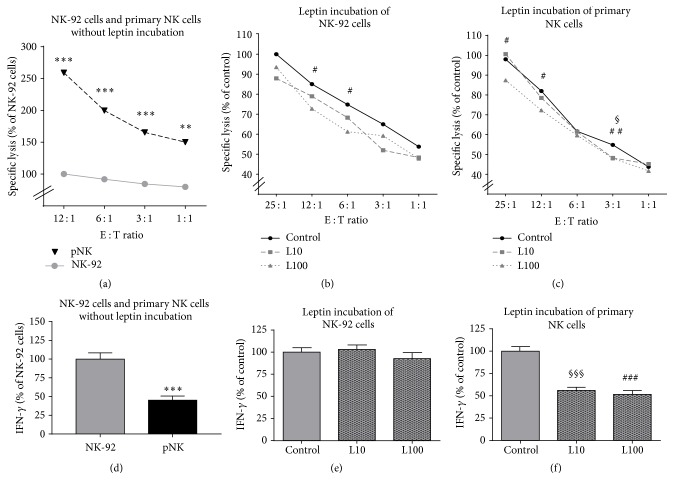
Cytotoxicity assay (a–c) and IFN-*γ* secretion (d–f) of NK-92 or primary NK (pNK) effector cells [E] against DLD-1 colon tumor cells target cells [T]. (a) Cytotoxicity assay of unstimulated NK-92 cells and unstimulated pNK cells. (b and c) Cytotoxicity assay of NK-92 cells or pNK cells after leptin incubation with 10 ng/mL (L10) or 100 ng/mL (L100) leptin for 72 h. Values are expressed as means ± SEM of at least three individual experiments with each *N* = 3-4 well. (d) Interferon–*γ* (IFN-*γ*) secretion of unstimulated NK-92 cells and unstimulated pNK cells. (e and f) IFN-*γ* secretion of NK-92 cells or pNK cells after leptin incubation. Values are expressed as means ± SEM at least in three individual experiments. ^*∗∗*^*P* < 0.01 and ^*∗∗∗*^*P* < 0.001, NK-92 cells compared to pNK cells; ^§^*P* < 0.05, ^§§§^*P* < 0.001, control group versus L10; ^#^*P* < 0.05, ^##^*P* < 0.01, and ^###^*P* < 0.001, control group versus L100.

To analyze the cytotoxicity of NK cells against colon tumor cells under the influence of leptin, europium release assays were performed using the NK-92 cell line or primary human NK cells as effector cells and DLD-1 cells as target cells. Primary NK cells showed a significantly higher cytotoxic potential against DLD-1 cells compared to NK-92 cells (Figure 1(a)). In contrast, IFN-*γ* secretion was significantly lower in primary NK cells compared to NK-92 cells when coincubated with DLD-1 cells (Figure 1(d)). Results of cytotoxicity assay demonstrated that leptin incubation (10 ng/mL and 100 ng/mL) for 72 h of both NK-92 cells and primary NK cells reduced the specific lysis of DLD-1 cells (Figures 1(b) and 1(c)). These effects were predominantly significant after incubation with 100 ng/mL leptin. Interestingly, IFN-*γ* secretion was not influenced by leptin incubation in NK-92 cells coincubated with DLD-1 cells (Figure 1(e)), whereas IFN-*γ* release of primary NK cells was significantly lower by leptin incubation compared to the unstimulated control (Figure 1(f)). In addition, experiments with short-term leptin incubation resulted in similar results: the cytotoxicity of NK-92 and primary NK cells against DLD-1 cells was significantly decreased by leptin incubation for 4 h. This was accompanied with a leptin-mediated reduction of IFN-*γ* secretion in primary NK cells, but not in NK-92 cells (data not shown).

### 3.2. Real-Time PCR Analyses of Leptin-Incubated NK-92 Cells

**Figure 2:**
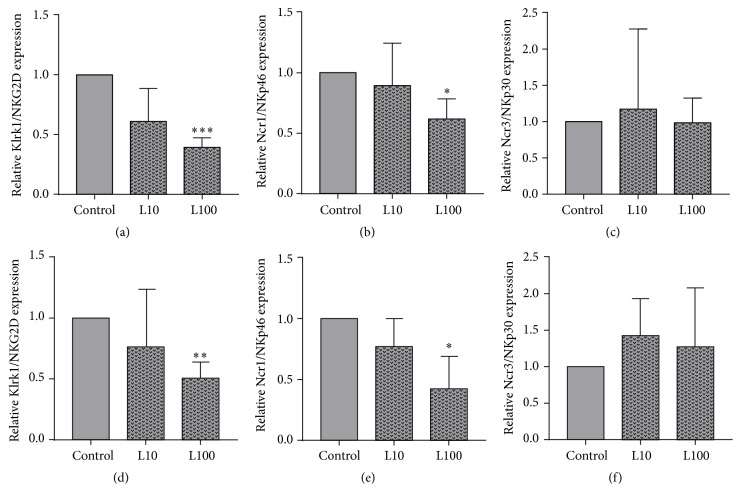
Influence of leptin on the mRNA expression of the activating NK cell receptors Klrk1/NKG2D (a, d), Ncr1/NKp46 (b, e), and Ncr3/NKp30 (c, f). NK-92 cells were incubated in the absence or presence of 10 ng/mL (L10) or 100 ng/mL (L100) leptin for 4 h (a–c) or 24 h (d–e). Results are expressed as the mean ± SEM fold-change compared to control cells, from three individual experiments with each *N* = 4 well. ^*∗*^*P* < 0.05, ^*∗∗*^*P* < 0.01, and ^*∗∗∗*^*P* < 0.001 compared to control cells.

To investigate the leptin effect on the mRNA expression of activating NK cell receptors, NK-92 cells were incubated in the absence or presence of 10 or 100 ng/mL leptin. The results show that incubation of NK-92 cells with 100 ng/mL leptin for 4 h or 24 h significantly decreased the Klrk1/NKG2D mRNA expression as well as the Ncr1/NKp46 mRNA expression, whereas expression levels of Ncr3/NKp30 mRNA were not influenced by leptin (Figure 2).

### 3.3. Dietary Intake and Anthropometric and Metabolic Parameters of Animal Experiments

The daily food intake of DIO rats was significantly lower compared to rats fed on a normocaloric diet. Due to the elevated energy content of the high-fat diet, total daily energy intake as well as the daily fat intake from fat was significantly increased in DIO rats. In contrast, DIO rats had a decreased energy intake from carbohydrates and proteins. The final body weight and visceral fat mass were significantly elevated in obese compared to the normal weight control group. The dietary intake and body weight changes were not influenced by tumor development. In addition, the visceral fat mass in AOM-treated control rats was decreased compared to NaCl-treated controls. Concentrations of triacylglycerol and total cholesterol showed no differences between the groups. In contrast, LDL cholesterol was significantly increased in serum of DIO rats independent of tumor development. Furthermore, HDL cholesterol was reduced in NaCl-treated but not in AOM-treated DIO rats compared to corresponding normal weight control group (Table 2).

### 3.4. Characterization of Tumor Development

**Figure 3:**
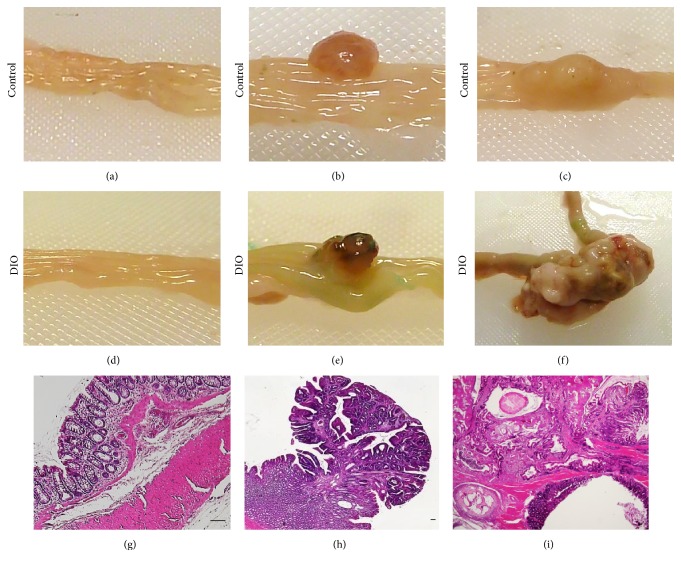
(a–f) Macroscopic observation of normal colon tissue, colorectal adenoma, and colorectal adenocarcinoma from azoxymethane- (AOM-) treated normal weight rats (a–c) as well as diet-induced obese (DIO) rats (d–f). (g–i) Representative histopathology of healthy colon tissue (g), colorectal adenoma (h), and colorectal adenocarcinoma ((i) scale bar, 100 *μ*m).

As expected, no colon tumors were detected in NaCl-treated normal weight and DIO rats. Number, distribution, pathohistology, weight, and size of colon tumors after 37 weeks of AOM treatment are shown in Table 3. Results demonstrate that more colon tumors had developed in AOM-treated DIO rats compared to normal weight control rats. In control rats, 50% of the tumors were located in the proximal part of the colon and 50% were located in the distal colon. Furthermore, 50% of all colon tumors were adenomas and 50% were carcinomas in normal weight control rats. In contrast, 33.3% of the tumors were located in the proximal colon and 66.6% were located in the distal part of the colon and 11% of all colon tumors were adenomas and 88.9% were carcinomas in DIO animals. The data also demonstrate a higher tumor size and tumor weight in DIO rats compared to the lean control group (Table 3).  Figure 3 illustrates representative macroscopic observations of normal colon tissue, colorectal adenoma, and colorectal adenocarcinoma from AOM-treated normal weight rats (Figures 3(a)–3(c)) and DIO rats (Figures 3(d)–3(f)), as well as representative histopathology of normal colon tissue, colorectal adenoma, and colorectal adenocarcinoma (Figures 3(g)–3(i)). No tumor metastases in secondary organs were detected macroscopically in the animals.

### 3.5. Analysis of Plasma Cytokines

**Figure 4:**
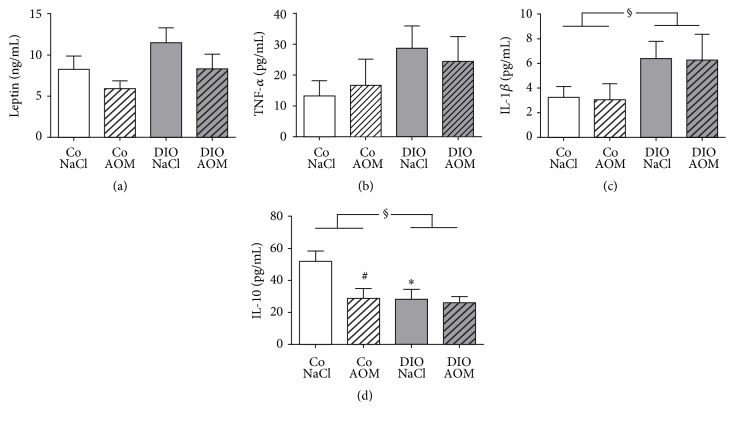
Plasma concentrations of leptin (a), TNF-*α* (b), IL-1*β* (c), and IL-10 (d) of normal weight [Co] and obese rats [DIO] treated with NaCl or azoxymethane (AOM). Data are expressed as mean ± SEM of *n* = 11–14 animals per group. ^#^*P* < 0.05 compared to NaCl-treated control group; ^*∗*^*P* < 0.05, compared to appropriate control group. ^§^*P* < 0.05, two-way-ANOVA, normal weight control groups compared to DIO groups.

Plasma leptin concentration was slightly elevated in DIO rats compared to normal weight rats (*P* = 0.06; Figure 4). In addition, the plasma levels of the proinflammatory cytokines TNF-*α* and IL-1*β* were higher in DIO rats compared to normal weight rats, with significant differences in IL-1*β* concentrations (Figures 4(b) and 4(c)). Results of leptin, TNF-*α*, and IL-1*β* concentrations appeared to be independent of AOM treatment. Furthermore, plasma concentration of IL-10 was reduced in NaCl-treated but not in AOM-treated DIO rats compared to the corresponding lean control group. Normal weight rats treated with AOM had significantly lower IL-10 plasma levels compared to the normal weight NaCl-treated group (Figure 4(d)).

### 3.6. Subset-Specific Alterations of Blood Leukocytes

**Figure 5:**
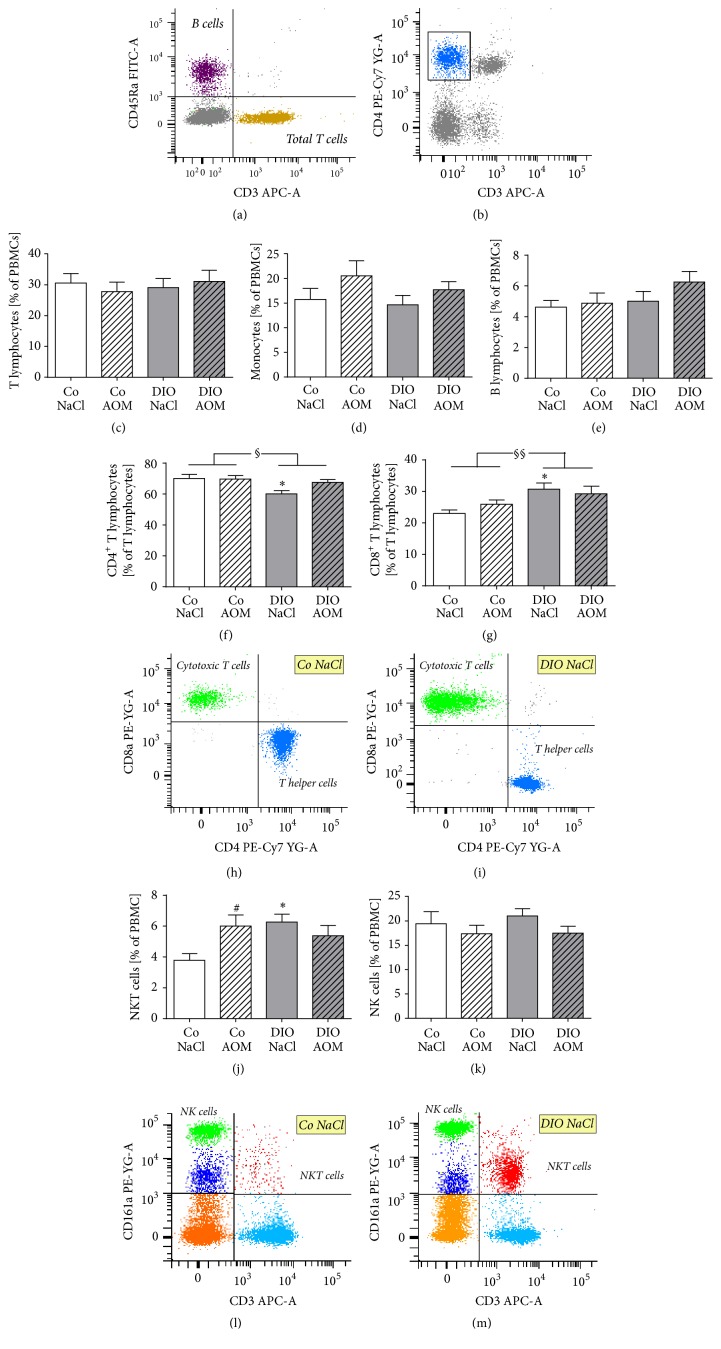
Exemplary FACS plots and frequencies of different leukocytes subset populations in peripheral blood of normal weight [Co] and obese [DIO] rats treated with NaCl or azoxymethane (AOM). Total T lymphocytes (a, c), monocytes (b, d), B lymphocytes (a, e), NKT cells (j, l, m), and NK cells (k, l, m) are demonstrated as percentage of peripheral blood mononuclear cells (PBMCs). NK cells are represented by cells with CD161a^bright^ and CD3^−^ expression and denoted with NK cells (green colored population). CD4+ T lymphocytes (f, h, i) and CD8+ T lymphocytes (g, h, i) are illustrated as percentage of total T lymphocytes. Differences in frequencies of CD4+ and CD8+ T lymphocytes (h, i) as well as differences in NKT cell frequency (l, m) in normal weight (h, l) and obese rats (i, m) are exemplary demonstrated. Data are expressed as mean ± SEM of *n* = 11–14 animals per group. ^#^*P* < 0.05 compared to NaCl-treated control group; ^*∗*^*P* < 0.05, compared to appropriate control group. ^§^*P* < 0.05, ^§§^*P* < 0.01, two-way-ANOVA, normal weight control groups compared to DIO groups.

Results of FACS analyses revealed no significant differences in the percentage of B lymphocytes, monocytes, and NK cells as well as of total T lymphocytes (Figures 5(c), 5(d), 5(e), and 5(k)). In contrast, significant changes were observed in T lymphocyte subsets: two-way ANOVA analyses revealed that DIO rats showed a significantly decreased level of T helper cells (CD4+) and an enhanced level of cytotoxic T cells (CD8+). In NaCl-treated animals, this effect was significant; the CD4/CD8 ratio changed from 3.05 in normal weight control rats to 2.00 in DIO rats (Figures 5(f) and 5(g)). In contrast, no significant differences were observed in levels of T helper cells or T helper cells comparing AOM-treated normal weight (CD4/CD8 ratio: 2.69) and DIO rats (CD4/CD8 ratio: 2.31). Different frequencies of CD4+ and CD8+ T lymphocytes in normal weight and obese control rats are demonstrated in exemplary FACS plots (Figures 5(h) and 5(i)). In addition, the NKT cell number was increased in AOM-treated lean animals compared to the NaCl-treated normal weight group (Figure 5(j)). Exemplary FACS plots demonstrate these changes in NKT cell frequencies comparing normal weight and DIO rats (Figures 5(l) and 5(m)). Furthermore, DIO rats showed a significantly elevated blood NKT cell number compared to normal weight rats after NaCl treatment but not after AOM treatment (Figure 5(j)).

### 3.7. Immunohistochemical Analyses

**Figure 6:**
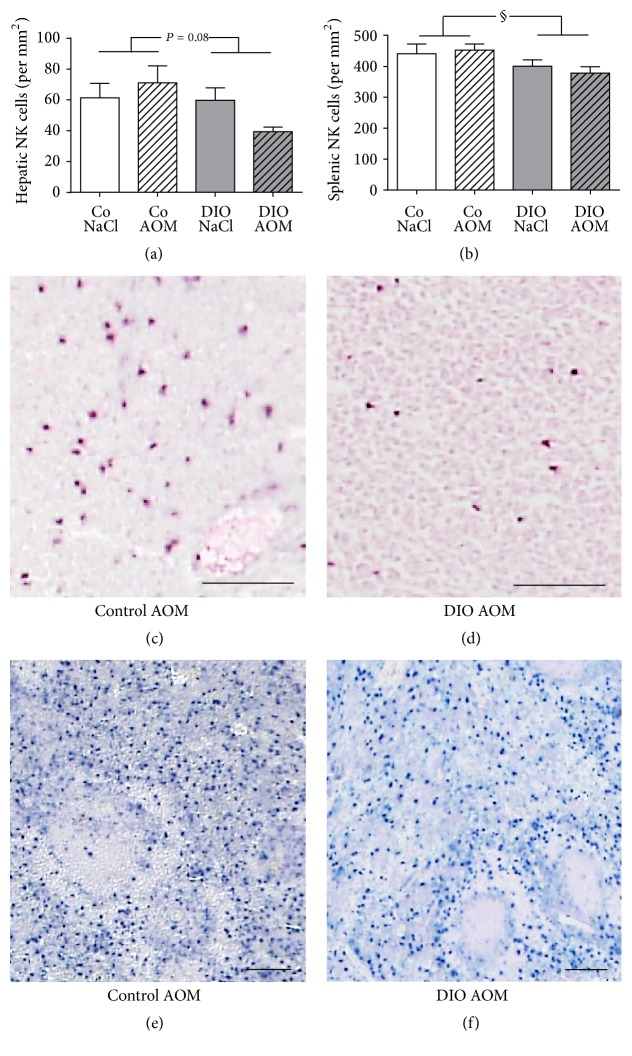
Numbers of NK cells in liver (a) and spleen (b) of normal weight [Co] and obese [DIO] rats treated with NaCl or azoxymethane (AOM). Data are expressed as mean ± SEM of *n* = 5 animals per group. ^§^*P* < 0.05, two-way-ANOVA, normal weight control groups compared to DIO groups. (c–f) Representative immunohistological detection of NK cells in liver tissue (red colored (c), (d)) and splenic tissue (blue colored; (e), (f)) in normal weight and obese rats treated with AOM (scale bar, 100 *μ*m).

Results of the immunohistological staining of tissue NK cells demonstrated decreased numbers of NK cells in liver and spleen of DIO rats compared to normal weight rats, with significant changes in the spleen (Figures 6(a) and 6(b)). Changes in tissue NK cell numbers were illustrated in representative sections of liver tissue (Figures 6(c) and 6(d)) as well as in splenic tissue (Figures 6(e) and 6(f)).

### 3.8. Molecular Investigations of Splenic and Hepatic Tissue

**Figure 7:**
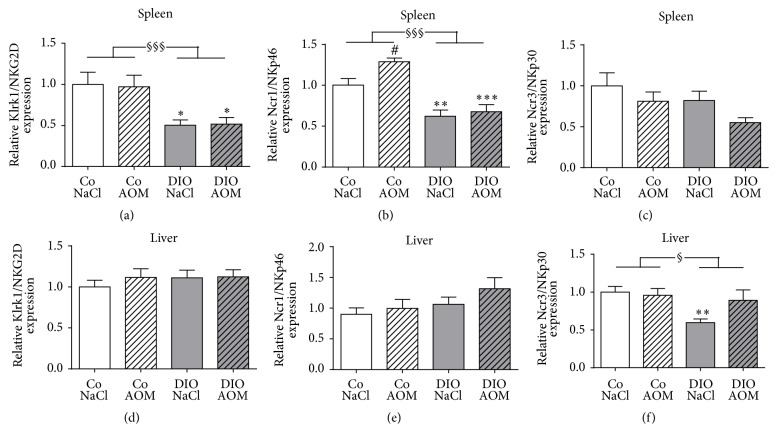
Splenic (a–c) and hepatic (d–f) mRNA expression of the NK cell activating receptors Klrk1/NKG2D (a, d), Ncr1/NKp46 (b, e), and Ncr3/NKp30 (c, f) in normal weight [Co] and obese [DIO] rats treated with NaCl or azoxymethane (AOM). Data are expressed as mean ± SEM of *n* = 11–14 animals per group. ^#^*P* < 0.05 compared to NaCl-treated control group; ^*∗*^*P* < 0.05, ^*∗∗*^*P* < 0.01, and ^*∗∗∗*^*P* < 0.001, compared to appropriate control group. ^§^*P* < 0.05, ^§§§^*P* < 0.001, and two-way-ANOVA, normal weight control groups compared to DIO groups.

To analyze the expression of activating NK cell receptors in spleen and liver, real-time RT-PCR was performed. In splenic tissue, expression levels of the activating receptors Klrk1/NKG2D and Ncr1/NKp46 were significantly lower in DIO rats treated with NaCl or AOM compared to the appropriate control group (Figures 7(a) and 7(b)). Furthermore, AOM treatment resulted in a significantly elevated Ncr1/NKp46 expression in normal weight rats but not in DIO rats (Figure 7(b)). Two-way ANOVA analyses revealed that Klrk1/NKG2D and Ncr1/NKp46 expression was significantly reduced in DIO rats compared to normal weight rats (Figures 7(a) and 7(b)). No significant changes between the groups were observed in expression levels of Ncr3/NKp30 (Figure 7(c)).

To evaluate tissue-specific gene expression levels, we further analyzed the expression of activating NK cell receptors in liver tissue. In contrast to the spleen, the Ncr1/NKp46 and Klrk1/NKG2D expression in the liver did not differ between all groups (Figures 7(d) and 7(e)). Obese NaCl-treated control rats had a significantly decreased Ncr3/NKp30 expression compared to the normal weight group, whereas no changes were observed comparing the AOM-treated animal groups (Figure 7(f)). Two-way ANOVA analyses revealed that the expression of Ncr3/NKp30 was significantly decreased in DIO groups compared to the lean animal groups (Figure 7(f)).

## 4. Discussion

Several studies demonstrated that obesity is associated with higher incidence and mortality rates for colon cancer [26]. Obesity is also associated with impaired immune functions. Beside the high amount of M2 macrophages secreting proinflammatory cytokines and an increase of proinflammatory T cells, neutrophils, and mast cells, the functionality of NK cells is disturbed in obese individuals [15, 17, 27]. As NK cells play an important role in identifying and killing tumor cells, we hypothesized that impaired NK cell functions may be one cause for the higher colon cancer risk in obesity.

Several former studies had already demonstrated that leptin influences NK cell cytotoxicity using different cell types as target cells [15, 24, 28–30]. Until now, no data existed about a leptin-mediated regulation on cytotoxic effects of NK cells against colon tumor cells. Results of the present investigations provide first evidence for a leptin-mediated decrease of cytotoxic activity of NK cells when they are coincubated with colon cancer cells. These results were found in NK-92 cells as well as in primary NK cells. Interestingly, the leptin-induced reduction of cytotoxicity was associated with a decreased IFN-*γ* secretion of primary NK cells but not of NK-92 cells. Furthermore, the data demonstrated that the basal lytic activity against colon cancer cells was significantly lower in NK-92 cells compared to primary NK cells. The NK-92 cell line exhibits phenotypical and functional characteristics of primary NK cells and is a well-established human cell line for investigations on NK cell physiology [31, 32]. However, our data elucidate that the application of findings on immortalized cell lines to primary cells freshly isolated from a metabolically defined milieu is quite limited. In contrast to our data of the cytotoxicity-reducing effect of leptin, several studies demonstrated that leptin increases the cytotoxic activity of NK cells [15, 24, 28, 29]. However, all these studies were performed using the human myelogenous leukemia line K652 or the murine lymphoma cell line YAC-1 as target cells but not on colon cancer cell lines. Therefore, the conflicting results of the leptin effect on the lytic activity of NK cells may result from the use of different tumor target cells for cytotoxicity assays as it was already demonstrated by Lamas et al. [30].

In addition to the decreasing leptin effect on IFN-*γ* secretion and cytotoxicity of NK cells, our results showed that leptin incubation also led to a reduced mRNA expression of the activating NK cell receptors NKp46 and NKG2D. Consequently, it can be assumed that leptin mediates the impaired NK cell activity against colon tumor cells via downregulation of activating NK cell receptors.

Beside leptin, concentrations of several metabolites, like glucose, insulin, and other adipocytokines, are changed in obese individuals and may also affect NK cell cytotoxicity. For example, earlier studies demonstrated that the adipocytokine adiponectin also influences NK cell function, although results were conflicting [16, 33, 34]. In addition, insulin-like growth factor 1 (IGF-1) was shown to modulate NK cell development and cytotoxicity [35]. Therefore, further studies are required to investigate the effects of other single obesity-related metabolites as well as adipocyte-conditioned medium as a mixture of components secreted by adipocytes on NK cell function to specify the meaning of obesity-related metabolites in influencing NK cell activity.

Obesity is associated with increased plasma leptin concentrations compared to normal weight individuals [22, 23]. Therefore, in our in vitro studies, leptin concentrations of 10 or 100 ng/mL were used to simulate physiological as well as pathologically elevated leptin concentrations in obesity. The results show that the inhibiting leptin effect on NK cell cytotoxicity as well as on expression of activating NK cell receptors reached significance solely using high leptin concentrations, like one would find in an obese condition. Studies in rodents and humans demonstrated that NK cell cytotoxicity in obesity was associated with higher serum leptin levels [14, 16, 18]. These data indicate that increased leptin concentrations in obesity leading to a reduced NK cell cytotoxicity may be one cause for the impaired tumor defense and increased tumor incidence in obese individuals. Results of this study also demonstrate that the decreased NK cell cytotoxicity by leptin is associated with a reduced IFN-*γ* secretion. Thus, leptin not only inhibits the direct lysis of target cells but also influences the antiviral and antitumor action by reducing the IFN-*γ* secretion of NK cells. As plasma and adipose tissue concentrations of proinflammatory cytokines, like IFN-*γ* and TNF-*α*, are elevated in obesity [36, 37], the leptin-mediated decrease of IFN-*γ* secretion by NK cells may be a local effect occurring in regions of NK cell defense against tumor cells. In this study, IFN-*γ* secretion was analyzed using NK cells incubated in medium containing 200 U/mL IL-2. Further studies using a more enhanced stimulation of NK cells by higher doses of IL-2 or the addition of IL-12, IL-15, or IL-18 as well as analyses of other cytokines released by NK cells after leptin incubation could complement the knowledge of the leptin effect on NK cell cytokine secretion.

To investigate the relevance of a disturbed NK cell functionality for the higher colon cancer risk in obesity, we performed an animal study using normal weight and diet-induced obese rats treated with AOM. Feeding a high-fat diet resulted in significantly elevated body weight and fat mass in the obese animals. The increase of the proinflammatory plasma cytokines TNF-*α* and IL-1*β* as well as the decrease of the anti-inflammatory plasma cytokine IL-10 displayed the low-grade inflammation status which is well known in obesity [38]. It is known that IL-1*β* inhibits maturation and functionality of NK cells [39, 40]. In addition, IL-10 was shown to increase NK cell proliferation, cytotoxicity, and cytokine secretion [41, 42]. Therefore, the higher IL-1*β* plasma concentrations and lower IL-10 plasma levels in obese animals found in this study may contribute to a suppression of NK cell killing activity against tumor cells and thereby the increased colon cancer incidence in obese rats.

Analyzes of the tumor development demonstrated that obese rats treated with AOM had a higher number, size, and weight of colon tumors and a higher rate of adenocarcinomas than adenomas. These data again confirm the elevated colon cancer risk and severity in obesity [9].

Results of this study showed that plasma leptin concentrations tended to be increased in obese AOM-treated rats compared to normal weight AOM-treated rats. With the knowledge of the inhibiting leptin effect of NK cell cytotoxicity against colon cancer cells especially in using leptin concentrations, it can be assumed that elevated plasma leptin levels in obese animals lead to a decrease of NK cell function which consequently may be one cause for the higher colon cancer risk and severity in obese rats.

Analyzing the blood leucocyte subset population, we found a decreased CD4/CD8 ratio in obese control rats compared to normal weight control rats. Previous studies provided conflicting results regarding T cell subpopulations in obesity [43–45]. CD8+ T cells are involved in initiating inflammatory cascades and maintenance of inflammatory response in obese adipose tissue [45]. The increase of CD8+ T cells in DIO rats probably plays a pathogenic role in the development of the obesity-associated inflammation. Interestingly, the CD4/CD8 ratio was not different comparing AOM-treated lean and obese rats. Obviously, the metabolic changes in tumor status prevented the shift of T helper cells to a higher number of cytotoxic T cells in obesity.

The reported role for NKT cells in obesity is controversial as other studies demonstrated no changes or a reduction of NKT cells in obese individuals [46, 47]. Our data show that the number of NKT cells was increased in AOM-treated normal weight animals compared to the NaCl-treated normal weight control group. Interestingly the NKT cell number was increased in the obese control group, but DIO rats failed to increase the circulating NKT cell number after AOM treatment. These results indicate an impaired NKT cell-mediated tumor-defending immune reaction in obesity.

In accordance with other investigations, the number of tissue NK cells in liver and spleen was reduced in DIO rats compared to lean animals in our study [29, 48, 49]. In addition, the mRNA expressions of the activating NK cell receptors NKG2D and NKp46 were significantly decreased in splenic tissue of obese animals. The NKp46 receptor mRNA expression was higher in AOM-treated lean rats compared to NaCl-treated lean rats. In contrast, DIO animals failed to react with an increase of the NKp46 receptor expression after AOM treatment. These data indicate an impaired NK cell activity against target cells in obesity. Interestingly, NK cell receptor expression pattern was different comparing splenic and liver tissue. These results demonstrate clear tissue-specific differences in NK cell characteristics. Nevertheless, with regard to the results of a decreased NKp46 and NKG2D receptor expression in NK-92 cells after leptin incubation, it can be assumed that the slightly increased plasma leptin levels in rats may contribute to the decreased mRNA expression of the activating NK cell receptors in spleen or liver of obese animals. This may be associated with a reduced defense of NK cells against tumor cells, resulting in a higher colon tumor risk. Further in vitro and in vivo studies are necessary to investigate the effects of leptin and other adipocytokines on the expression of activating NK cell receptors and involved leptin-mediated signaling cascades to influence NK cell activity.

In addition, future investigations have to analyze the NK cell receptor expression specifically in colon tumor tissue to compare the activation status of NK cells in normal weight and obese individuals in terms of direct cellular tumor defense and consecutive tumor development. In addition, investigations with normal weight and obese patients contracted or noncontracted to colon carcinoma have to be performed to characterize blood and tumor tissue NK cell activity in relation to BMI in human individuals.

Several studies demonstrated that weight loss in obesity was associated with a reduction of colon cancer incidence and mortality [50–52]. In addition, investigations provided evidence that impaired NK cell activity in obesity can be improved by reduction of fat and energy intake, weight stability, and weight loss after bariatric surgery or a moderate dietary and exercise program [20, 21, 53–56]. Therefore, prevention of overweight and fat mass reduction in obesity by physical training or caloric restriction can attenuate the colon cancer risk by improving the NK cell functionality.

The relevance of NK cells in the defense against colon tumor development has been well investigated [57, 58]. Mori et al. revealed evidence that the decreased NK cell function in obesity was associated with a more serious tumor development [59]. Accordingly, the results of the in vitro and in vivo investigations of this study indicate a clear association of the disturbed NK cell functionality and the higher colon tumor risk in obesity for the first time.

## Figures and Tables

**Table 1:** Characteristics of the specific primers used for real-time RT-PCR analysis.

Gene	Protein	Species	Sequence	bp	NCBI reference
Actb	Beta-actin	Human	Fw: GACGACATGGAGAAAATCTG	131	NM_001101
			Rv: ATGATCTGGGTCATCTTCTC		
Ppia	CypA	Rat	Fw: GTGTTCTTCGACATCACG	92	NM_017101
			Rv: TGTCTGCAAACAGCTCGAAG		
Klrk1	NKG2D	Rat	Fw: TTAATGAGAACAAAGCCTGG	199	NM_133512
			Rv: GTTAACTCGTTGGGTGATAG		
Klrk1	NKG2D	Human	Fw: AGGACAAAATGACCAAAGAC	135	NM_007360
			Rv: CTTGGGGATATCTGAATTGC		
Ncr1	NKp46	Rat	Fw: AGACCCTGTTTCTTCTCTTG	197	NM_057199
			Rv: TGAGCTTCTCATGATCCTTC		
Ncr1	NKp46	Human	Fv: CAGAAATGTATGACACACCC	119	NM_001145457
			Rv: CTTGAGCAGTAAGAACATGC		
Ncr3	NKp30	Rat	Fw: AAGAGCCTCCTCAACAAG	94	NM_181822
			Rv: AGAAAGCTGAGGGCATAG		
Ncr3	NKp30	Human	Fw: ATATGCCAAATCTACTCTCTCC	200	NM_001145466
			Rv: TCTGATCCTTCCCATTTCTC		

**Table 2:** Anthropometric, nutritional, and metabolic data in normal weight (control) or diet-induced obese (DIO) rats treated with NaCl or azoxymethane (AOM).

	Control		DIO		Two-way ANOVA
	NaCl	AOM	NaCl	AOM	*P* value
*Anthropometric indices*					
Body weight (g)	700.4 ± 25.0	672.3 ± 33.7	810.1 ± 28.5^*∗∗*^	780.2 ± 38.5^*∗*^	0.0011^§§^
Visceral fat mass (g)	51.5 ± 5.0	34.7 ± 5.9^#^	72.3 ± 7.6^*∗*^	64.1 ± 10.5^*∗*^	0.0009^§§§^
*Daily dietary intake*					
Food intake (g)	24.7 ± 1.6	99.6 ± 4.2	85.0 ± 5.0	20.3 ± 0.6^*∗*^	0.0041^§§^
Total energy intake (kJ)	364.6 ± 23.4	350.8 ± 14.2	445.8 ± 26	444.5 ± 12.6^*∗∗*^	0.0007^§§§^
Energy intake from carbohydrates (KJ)	240.7 ± 15.5	231.5 ± 9.2	102.6 ± 5.9^*∗∗*^	102.1 ± 2.9^*∗∗∗*^	0.0001^§§§^
Energy intake from fat (KJ)	36.4 ± 2.5	35.2 ± 1.3	267.5 ± 15.5^*∗∗∗*^	267.1 ± 7.5^*∗∗∗*^	0.0001^§§§^
Energy intake from proteins (KJ)	87.5 ± 5.4	84.1 ± 0.4	75.8 ± 4.6	75.8 ± 2.1	0.0237^§^
*Metabolic parameters*					
Triacylglycerol (mmol/L)	2.4 ± 0.3	2.0 ± 0.2	2.8 ± 0.2	2.5 ± 0.3	0.1025
Total cholesterol (mmol/L)	2.3 ± 0.2	1.9 ± 0.2	1.9 ± 0.1^*∗*^	2.0 ± 0.1	0.2738
LDL (*μ*mol/L)	258.6 ± 0.1	259.6 ± 1.0	359.3 ± 19.0^*∗∗∗*^	349.0 ± 19.0^*∗∗∗*^	0.0001^§§§^
HDL (mmol/L)	2.0 ± 0.1	1.7 ± 0.2	1.5 ± 0.1^*∗∗*^	1.5 ± 0.1	0.0045^§§^

All data are expressed as mean ± SEM of *n* = 11–14 animals per group. Data of kilocalories (kcal) are given in parentheses. ^#^*P* < 0.05, compared to NaCl-treated control group; ^*∗*^*P* < 0.05, ^*∗∗*^*P* < 0.01, and ^*∗∗∗*^*P* < 0.001, compared to appropriate normal weight control; ^§^*P* < 0.05, ^§§^*P* < 0.01, and ^§§§^*P* < 0.001, normal weight control groups compared to DIO groups.

**Table 3:** Characterization of colon tumors in normal weight (control) or diet-induced obese (DIO) rats treated with azoxymethane (AOM).

	Control	DIO
*Tumor number and localization*		
Animals per group	12	11
Total tumor number	6	9
Proximal colon tumors	3	3
Distal colon tumors	3	6
*Tumor type*		
Adenoma	3	1
Adenocarcinoma	3	8
*Tumor parameters*		
Tumor size (mm^2^)^*∗*^	25.88 ± 12.87	47.72 ± 13.28
Tumor weight (mg)^*∗*^	6.81 ± 4.79	14.53 ± 4.66

^*∗*^Data are expressed as mean ± SEM.

## References

[1] WHO (2015). *Obesity and Overweight*.

[2] Dobbins M., Decorby K., Choi B. C. (2013). The Association between obesity and cancer risk: a meta-analysis of observational studies from 1985 to 2011. *ISRN Preventive Medicine*.

[3] Calle E. E., Rodriguez C., Walker-Thurmond K., Thun M. J. (2003). Overweight, obesity, and mortality from cancer in a prospectively studied cohort of U.S. Adults. *The New England Journal of Medicine*.

[4] Wolin K. Y., Carson K., Colditz G. A. (2010). Obesity and cancer. *Oncologist*.

[5] Alemán J. O., Eusebi L. H., Ricciardiello L., Patidar K., Sanyal A. J., Holt P. R. (2014). Mechanisms of obesity-induced gastrointestinal neoplasia. *Gastroenterology*.

[6] Keum N., Greenwood D. C., Lee D. H. O. (2015). Adult weight gain and adiposity-related cancers: a dose-response meta-analysis of prospective observational studies. *Journal of the National Cancer Institute*.

[7] Larsson S. C., Wolk A. (2007). Obesity and colon and rectal cancer risk: a meta-analysis of prospective studies. *The American Journal of Clinical Nutrition*.

[8] Campbell P. T., Newton C. C., Dehal A. N., Jacobs E. J., Patel A. V., Gapstur S. M. (2012). Impact of body mass index on survival after colorectal cancer diagnosis: the Cancer Prevention Study-II Nutrition Cohort. *Journal of Clinical Oncology*.

[9] Bardou M., Barkun A. N., Martel M. (2013). Obesity and colorectal cancer. *Gut*.

[10] Nistal E., Fernández-Fernández N., Vivas S., Olcoz J. L. (2015). Factors determining colorectal cancer: the role of the intestinal microbiota. *Frontiers in Oncology*.

[11] Stewart C. A., Vivier E., Colonna M. (2006). Strategies of natural killer cell recognition and signaling. *Current Topics in Microbiology and Immunology*.

[12] Yaqoob P., Newsholme E. A., Calder P. C. (1994). Inhibition of natural killer cell activity by dietary lipids. *Immunology Letters*.

[13] Jeffery N. M., Sanderson P., Newsholme E. A., Calder P. C. (1997). Effects of varying the type of saturated fatty acid in the rat diet upon serum lipid levels and spleen lymphocyte functions. *Biochimica et Biophysica Acta (BBA)—Lipids and Lipid Metabolism*.

[14] Smith A. G., Sheridan P. A., Harp J. B., Beck M. A. (2007). Diet-induced obese mice have increased mortality and altered immune responses when infected with influenza virus. *Journal of Nutrition*.

[15] Nave H., Mueller G., Siegmund B. (2008). Resistance of Janus kinase-2 dependent leptin signaling in natural killer (NK) Cells: a novel mechanism of NK cell dysfunction in diet-induced obesity. *Endocrinology*.

[16] O'Shea D., Cawood T. J., O'Farrelly C., Lynch L. (2010). Natural killer cells in obesity: impaired function and increased susceptibility to the effects of cigarette smoke. *PLOS ONE*.

[17] Viel S., Besson L., Charrier E. (2016). Alteration of Natural Killer cell phenotype and function in obese individuals. *Clinical Immunology*.

[18] Laue T., Wrann C. D., Hoffmann-Castendiek B., Pietsch D., Hübner L., Kielstein H. (2015). Altered NK cell function in obese healthy humans. *BMC Obesity*.

[19] Lautenbach A., Wrann C. D., Jacobs R., Müller G., Brabant G., Nave H. (2009). Altered phenotype of NK cells from obese rats can be normalized by transfer into lean animals. *Obesity*.

[20] Moulin C. M., Marguti I., Peron J. P. S., Halpern A., Rizzo L. V. (2011). Bariatric surgery reverses natural killer (NK) cell activity and NK-related cytokine synthesis impairment induced by morbid obesity. *Obesity Surgery*.

[21] Jahn J., Spielau M., Brandsch C. (2015). Decreased NK cell functions in obesity can be reactivated by fat mass reduction. *Obesity*.

[22] Shah N. R., Braverman E. R. (2012). Measuring adiposity in patients: the utility of body mass index (BMI), percent body fat, and leptin. *PLoS ONE*.

[23] Considine R. V., Caro J. F. (1996). Leptin in humans: current progress and future directions. *Clinical Chemistry*.

[24] Wrann C. D., Laue T., Hübner L. (2012). Short-term and long-term leptin exposure differentially affect human natural killer cell immune functions. *American Journal of Physiology - Endocrinology and Metabolism*.

[25] Pfaffl M. W. (2001). A new mathematical model for relative quantification in real-time RT-PCR. *Nucleic Acids Research*.

[26] Basen-Engquist K., Chang M. (2011). Obesity and cancer risk: recent review and evidence. *Current Oncology Reports*.

[27] Gerriets V. A., MacIver N. J. (2014). Role of T cells in malnutrition and obesity. *Frontiers in Immunology*.

[28] Zhao Y., Sun R., You L., Gao C., Tian Z. (2003). Expression of leptin receptors and response to leptin stimulation of human natural killer cell lines. *Biochemical and Biophysical Research Communications*.

[29] Tian Z., Sun R., Wei H., Gao B. (2002). Impaired natural killer (NK) cell activity in leptin receptor deficient mice: leptin as a critical regulator in NK cell development and activation. *Biochemical and Biophysical Research Communications*.

[30] Lamas B., Goncalves-Mendes N., Nachat-Kappes R. (2013). Leptin modulates dose-dependently the metabolic and cytolytic activities of NK-92 cells. *Journal of Cellular Physiology*.

[31] Gong J.-H., Maki G., Klingemann H.-G. (1994). Characterization of a human cell line (NK-92) with phenotypical and functional characteristics of activated natural killer cells. *Leukemia*.

[32] Klingemann H., Boissel L., Toneguzzo F. (2016). Natural killer cells for immunotherapy—advantages of the NK-92 cell line over blood NK cells. *Frontiers in Immunology*.

[33] Wilk S., Jenke A., Stehr J. (2013). Adiponectin modulates NK-cell function. *European Journal of Immunology*.

[34] Kim K.-Y., Kim J. K., Han S. H. (2006). Adiponectin is a negative regulator of NK cell cytotoxicity. *The Journal of Immunology*.

[35] Ni F., Sun R., Fu B. (2013). IGF-1 promotes the development and cytotoxic activity of human NK cells. *Nature Communications*.

[36] Lucas R., Parikh S. J., Sridhar S. (2013). Cytokine profiling of young overweight and obese female African American adults with prediabetes. *Cytokine*.

[37] Dandona P., Weinstock R., Thusu K., Abdel-Rahman E., Aljada A., Wadden T. (1998). Tumor necrosis factor-*α* in sera of obese patients: fall with weight loss. *Journal of Clinical Endocrinology and Metabolism*.

[38] Cooke A. A., Connaughton R. M., Lyons C. L., McMorrow A. M., Roche H. M. (2016). Fatty acids and chronic low grade inflammation associated with obesity and the metabolic syndrome. *European Journal of Pharmacology*.

[39] Elkabets M., Ribeiro V. S. G., Dinarello C. A. (2010). IL-1*β* regulates a novel myeloid-derived suppressor cell subset that impairs NK cell development and function. *European Journal of Immunology*.

[40] Ambrosini P., Loiacono F., Conte R. (2015). IL-1beta inhibits ILC3 while favoring NK-cell maturation of umbilical cord blood CD34(+) precursors. *European Journal of Immunology*.

[41] Lauw F. N., Pajkrt D., Hack C. E., Kurimoto M., Van Deventer S. J. H., Van der Poll T. (2000). Proinflammatory effects of IL-10 during human endotoxemia. *The Journal of Immunology*.

[42] Cai G., Kastelein R. A., Hunter C. A. (1999). IL-10 enhances NK cell proliferation, cytotoxicity and production of IFN-*γ* when combined with IL-18. *European Journal of Immunology*.

[43] Tanaka S.-I., Isoda F., Ishihara Y., Kimura M., Yamakawa T. (2001). T lymphopaenia in relation to body mass index and TNF-*α* in human obesity: adequate weight reduction can be corrective. *Clinical Endocrinology*.

[44] O'Rourke R. W., Kay T., Scholz M. H. (2005). Alterations in T-cell subset frequency in peripheral blood in obesity. *Obesity Surgery*.

[45] Nishimura S., Manabe I., Nagasaki M. (2009). CD8+ effector T cells contribute to macrophage recruitment and adipose tissue inflammation in obesity. *Nature Medicine*.

[46] Nieman D. C., Henson D. A., Nehlsen-Cannarella S. L. (1999). Influence of obesity on immune function. *Journal of the American Dietetic Association*.

[47] Lynch L., O'Shea D., Winter D. C., Geoghegan J., Doherty D. G., O'Farrelly C. (2009). Invariant NKT cells and CD1d^+^ cells amass in human omentum and are depleted in patients with cancer and obesity. *European Journal of Immunology*.

[48] Lynch L. A., O'Connell J. M., Kwasnik A. K., Cawood T. J., O'Farrelly C., O'Shea D. B. (2009). Are natural killer cells protecting the metabolically healthy obese patient?. *Obesity*.

[49] Lo C. K., Lam Q. L., Yang M. (2009). Leptin signaling protects NK cells from apoptosis during development in mouse bone marrow. *Cellular and Molecular Immunology*.

[50] Afshar S., Kelly S. B., Seymour K., Lara J., Woodcock S., Mathers J. C. (2014). The effects of bariatric surgery on colorectal cancer risk: systematic review and meta-analysis. *Obesity Surgery*.

[51] Williamson D. F., Pamuk E., Thun M., Flanders D., Byers T., Heath C. (1995). Prospective study of intentional weight loss and mortality in never-smoking overweight US white women aged 40-64 years. *American Journal of Epidemiology*.

[52] Parker E. D., Folsom A. R. (2003). Intentional weight loss and incidence of obesity-related cancers: the Iowa Women's Health Study. *International Journal of Obesity and Related Metabolic Disorders*.

[53] Shade E. D., Ulrich C. M., Wener M. H. (2004). Frequent intentional weight loss is associated with lower natural killer cell cytotoxicity in postmenopausal women: possible long-term immune effects. *Journal of the American Dietetic Association*.

[54] Barone J., Hebert J. R. (1988). Dietary fat and Natural Killer cell activity. *Medical Hypotheses*.

[55] Lamas O., Martínez J. A., Marti A. (2004). Energy restriction restores the impaired immune response in overweight (cafeteria) rats. *Journal of Nutritional Biochemistry*.

[56] Wing E. J., Stanko R. T., Winkelstein A., Adibi S. A. (1983). Fasting-enhanced immune effector mechanisms in obese subjects. *The American Journal of Medicine*.

[57] Coca S., Perez-Piqueras J., Martinez D. (1997). The prognostic significance of intratumoral natural killer cells in patients with colerectal carcinoma. *Cancer*.

[58] Wang Y., Sun J., Ma C. (2016). Reduced expression of galectin-9 contributes to a poor outcome in colon cancer by inhibiting NK cell chemotaxis partially through the Rho/ROCK1 signaling pathway. *PLoS ONE*.

[59] Mori A., Sakurai H., Choo M.-K. (2006). Severe pulmonary metastasis in obese and diabetic mice. *International Journal of Cancer*.

